# Diagnosis and Monitoring of Prostatic Lesions: A Comparison of Three Modalities: Multiparametric MRI, Fusion MRI/Transrectal Ultrasound (TRUS), and Traditional TRUS

**DOI:** 10.7759/cureus.702

**Published:** 2016-07-18

**Authors:** Antoinette Birs, Peter H Joyce, Zoran J Pavlovic, Alexander Lim

**Affiliations:** 1 University of Central Florida College of Medicine

**Keywords:** active surveillance, prostate cancer, transrectal ultrasound, prostate-specific antigen, digital rectal exam, multiparametric mri

## Abstract

Purpose: Transrectal ultrasound (TRUS) has been the gold standard of imaging for diagnosing prostate cancer for decades but is plagued by user error and undersampling. We aim to explore imaging modalities that are now being used in combination or alone for screening, diagnosis, and/or active surveillance of prostate cancer.

Methods: A PubMed literature search was performed to include articles published up to April 2016. Data were extracted and analyzed.

Results: Several large-scale studies have found an increased cancer detection rate in MRI-targeted lesions with an improved ability to target anterior lesions as well as an increased cancer detection in high-risk cancers using fusion platforms vs TRUS alone.

Conclusions: To date, there have been few head-to-head trials to directly compare the use of multiparametric MRI (mpMRI), transrectal ultrasound, and MRI-ultrasound fusion modalities for accurate and reliable detection, active surveillance, or biopsy procedure success rates. Further investigation utilizing these modalities are needed before they can be relied upon in active surveillance management, although mpMRI appears to be currently the most reliable in monitoring and diagnosing prostate lesions.

## Introduction and background

Prostate-specific antigen (PSA) has been the gold standard screening test for prostate cancer since its approval by the FDA in 1986 [1]. Its original use was for the surveillance of men who had known prostate cancer. This simple serum analysis expanded its utility in 1994 when it was approved as a screening test when combined with digital rectal exam (DRE) for screening asymptomatic men [1].

Prostate cancer is the most common noncutaneous cancer in America and the most common cancer in men; The American Cancer Society estimates that there will be 180,890 new cases of prostate cancer in the United States in 2016 [2]. As recommended by the European Urologic Association, management of patients with a high PSA and an abnormal finding on DRE necessitates a 12-core systematic ultrasound-guided transrectal biopsy in search of tissue confirmation of cancer. Transrectal biopsies are invasive procedures associated with complications, such as bleeding, pain, and infection, and should be avoided until absolutely necessary. Increasing numbers of abnormal biopsies may lead to increasing numbers of patients undergoing aggressive management.

Evidence began to accumulate against the utility of using PSA levels to screen for cancer; detecting prostate cancer early may not reduce the chance of dying from prostate cancer and the screening may give false positive or negative results. A large-scale population-based trial completed in 2006, The Prostate, Lung, Colorectal and Ovarian Cancer Screening Trial, found that men undergoing annual PSA and DRE screening had the same rate of deaths from disease as those in the control group while those in the screened group had a higher incidence of prostate cancer [3]. The argument against screening and aggressive treatment in those with positive screenings lies in the natural history of this disease process, which can be indolent; many men may die with prostate cancer without ever treating it or knowing their diagnosis [4].

One potential shortcoming in the PSA screening tool lies in the fact that the screening value cutoff of 4 ng/mL as the reference range for PSA was originally standardized using the PSA levels in Caucasian males. Older age, African-American race, and a positive family history are strong risk factors for prostate cancer. African-Americans are 56% more likely to develop prostate cancer than white males and 2.5 times as likely to die from it; several studies similarly determined that PSA levels in black men are higher than those in white men regardless of age or presence of prostate cancer [5]. As there is variability in the “normal range” of different age groups and races, use of standardized PSA measurements specific for age and race may improve the specificity of the serum PSA test. Despite an effort to create better reference standards, acute or chronic processes, such as prostatitis, benign prostatic hyperplasia, and other factors, may cause unpredictable elevations of PSA.

Yearly PSA screening has fallen out of favor with most healthcare professionals and national healthcare organizations for the above-stated reasons; however, there is still delay in the adoption of these guidelines in general practice as insurance companies and Medicare are still covering annual PSA screenings in men over 50 [1]. Yearly PSA screenings were given a grade D recommendation, meaning that there is moderate or high certainty that the service has no benefit or that the harms outweigh the benefits, by the 2012 United States Preventative Services Task Force. Screening modalities need to be improved to prevent deaths from prostate cancer and also take into consideration the serious treatment complications incurred by patients with low-grade prostate cancer. The US Preventive Services Task Force has analyzed the data from the Prostate, Lung, Colorectal, and Ovarian Cancer Screening Trial and the European Randomized Study of Screening for Prostate Cancer (ERSPC) and found that for every 1,000 men aged 55 to 69 years old, screened every one to four years for a decade, there would be one death at most from prostate cancer that would be avoided; however, it would result in the diagnosis of 110 men with prostate cancer and of those 110, 50 would have a complication from treatment, such as erectile dysfunction, urinary incontinence, or serious cardiovascular events [6].  These data indicate that for prostate cancer, an aggressive approach to treatment may do more harm than good.

We look to discuss other imaging modalities that may be used for screening, diagnosis, and active surveillance of prostate cancer that may result in more sensitive testing and lower the false positives or better classify and/or monitor low disease burden. This review will discuss the use of multiparametric MRI, transrectal ultrasound, and MRI-ultrasound fusion modalities in prostate cancer screening, prevention, and diagnosis.  

## Review

### Transrectal ultrasound (TRUS)

The advent of prostatic biopsy via a transrectal approach served as a welcomed replacement for the previous percutaneous transperineal approach, which was both technically challenging for the practitioner and painful for the patient [7]. Current guidelines recommend prostatic biopsy for all patients with elevated serum prostate-specific antigen (PSA) or abnormal prostate morphology on digital rectal exam. Standard practice for transrectal ultrasound (TRUS)-guided biopsy involves the utilization of 10-12 core needle (16-18 gauge) biopsies; however, there is not an established ideal number of biopsies [7-8]. Initially, in 1989, Hodge introduced the practice of a six-core biopsy, but this method was later found to miss 22-30% of prostatic cancers. Since its introduction, the number of core needle biopsies has been on the rise to decrease sampling errors and increase prostatic cancer detection rates [7]. Extended biopsies have been found to improve the concordance of Gleason scores between prostatic biopsies and prostatectomy [9]. In contrast, Rodriguez-Patrón, et al. determined that in a comparison of 221 patients undergoing six-core versus ten-core biopsies, only 2.25% of patients benefited from the augmented number of biopsies [8].

Prostate cancer (PCa) is often multifocal and microscopic; thus, detection can be quite difficult with limitations in the sampling number [10]. TRUS-guided biopsies often fail to detect cancerous lesions, misclassify the cancer risk, and have been found to have sensitivity for detection as low as 60% [7, 11]. A study by Ouzzane, et al. found up to 46% of 12-core biopsies in their trial had missed anteriorly located cancerous lesions [12-13]. Hu, et al. determined that transperineal prostate mapping (TPM) was more accurate versus five traditional TRUS-guided strategies at detecting and excluding lesions of tumor volume thresholds of 0.2 mL and 0.5 mL [11]. Additionally, TRUS-biopsy failed to detect 30-40% of lesions > 0.2 mL compared to 5% missed in TPM [11]. Schulte, et al. determined that in men classified with unilateral disease on extended 12-core biopsy, only 40.4% had true unilateral disease when reevaluated on radical prostatectomy [14]. In addition, the transperineal approach has no advantage over the transrectal approach with respect to the determination of tumor laterality [15].

While TRUS-guided prostatic biopsies have become a welcome replacement for the transperineal approach, this technique is not without disadvantages. TRUS-guided biopsy of the prostate involves penetration through the rectal mucosa, leading to potential patient risk and complications. Post-biopsy complications include sepsis, hospitalization, bacteriuria, bacteremia, and acute urinary retention [16]. Peri-biopsy prophylactic fluoroquinolone has been found to decrease infectious complication rates, but there has been a development of increasing antimicrobial resistance [17]. In a five-year study of 12,968 TRUS-guided biopsies, 32 ( 0.25%) patients died within 30 days post-biopsy and 1,266 (9.76%) patients suffered from post-biopsy voiding difficulty [9]. When compared to transrectal biopsy, transperineal biopsy is associated with a higher prevalence of acute urinary retention and lower burden of hospitalization and sepsis [16]. However, compared to lung, colorectal, and ovarian sampling in screening, prostate biopsy demonstrates a very low risk and no evidence of excess mortality post-biopsy [18].

The accuracy of TRUS-guided biopsy can be improved with anterior sampling, but this approach requires the utilization of transperineal template prostate mapping, which involves added patient discomfort and additional procedure risk [7, 11]. New literature suggests a change in management culture from the removal of lesions towards an active surveillance of prostate cancer [4]. To achieve this, the reliability of TRUS-guided biopsy must be increased, such that the same area can be reliably surveyed multiple times. Suggested strategies to address this include extended biopsies, transperineal saturation biopsies, and linkage of prostate cancer biomarkers, such as TMPRSS2-ERG, to ultrasound imaging [7]. New advances in technology, including contrast-enhanced transrectal ultrasound (CE-TRUS) and real-time sonoelastography (RTE), have shown promise, allowing for targeted biopsies and improving PCa detection [19]. In addition, ultrasound may appear sonographically normal while containing neoplastic foci [5].

### Multiparametric MRI

Magnetic resonance imaging (MRI) has evolved from a concept rooted in quantum mechanics to a crucial and relevant healthcare imaging modality. The ability of an MRI to display and differentiate minor soft tissue details have aided in the diagnosis and treatment of many life-threatening diseases. Moreover, MRI has solidified its role in cancer diagnosis by being a useful tool for early stage detection, decreasing overall cancer mortality. Prostate cancer (PCa) diagnosis and treatment have especially benefitted from advancements in the resolution and practicality of MRI, particularly multiparametric MRI (mpMRI). MpMRI is a three component MRI modality consisting of regular T2-weighted imaging (T2WI), diffusion-weighted imaging (DWI), and dynamic contrast-enhanced imaging (DCEI).

T2-weighted imaging, which highlights tissues containing a high water content (such as CSF), provides excellent differentiation of anatomical zones of the prostate, such as the peripheral zone (PZ), displayed as a relatively increased signal intensity, the central zone (CZ), displayed as relatively decreased signal intensity, and the transitional zone (TZ), exhibiting a heterogeneous, often swirled, signal intensity [20]. These images correlate with prostate cancer Gleason scores in that higher scores exhibit decreased signal intensity. Disadvantages of T2WI are false positives arising from more benign processes, such as acute and chronic prostatitis, atrophy, scars, post-irradiation or hormonal treatment effects, hyperplasia, and post-biopsy hemorrhage. In addition, PCa can often be difficult to detect in the TZ, but a TZ diagnosis error can be reduced by looking for details in lesion abnormalities, such as homogenously decreased signal intensity, poorly defined and irregular lesion borders, invasion into the urethra or fibromuscular stroma, and lenticular lesion shape [21]. 

DWI takes advantage of the physics concept of Brownian motion, defined as the random motion of particles contained in a defined space and suspended in a fluid (i.e., gas or liquid) that can be affected by random collisions with other particles. Within tissue, the Brownian motion refers to the random movement of water molecules in confined intracellular or extracellular spaces. Moreover, b-value selection (b-value refers to the strength and timing of gradients, defined by pulses consisting of varying magnitude and duration separated by time intervals, used to create diffusion-weighted images) has proved to be crucial in detecting PCa lesions. Traditionally, a maximal b-value of 1,000 s/mm^2^ has been used, although recent studies show that using a b-value of 2,000 s/mm^2^ can increase the accuracy of PCa detection in the PZ and TZ by lowering background noise [22-23]. Additionally, the apparent diffusion coefficient (ADC) can be calculated by measuring signal intensities at multiple b-values to quantify water movement restriction. ADC exhibits an inverse relationship between quantitative ADC and the Gleason score indicating utility in the predictive value of ADC for tumor aggressiveness; however, more studies need to be performed to confirm ADC's predictive strength in comparison to the Gleason score [24]. The limitations of DWI arise with low signal-to-noise ratios and image distortion, which worsens with higher b-values indicating that image optimization will correlate with user determined b-values [25].

DCE-MRI consists of a series of rapid T1-weighted sequences imaging the entire prostate before and after a bolus injection of gadolinium chelate at 2-4 mL per second [26]. This imaging modality utilizes principles of contrast kinetics and incorporates the signal increase/peak upon injection of contrast and follows the kinetics of contrast diffusion out of various areas of tissue. As cancers have increased vascularity, the theory is that cancerous lesions will reach higher peak signals and also have delayed dissipation of the contrast from the lesion. DCE-MRI, therefore, is useful in identifying the previously mentioned signal differences in lesions that are suspected to be cancerous. However, DCE-MRI signals can vary greatly and can, at times, overlap with the signals that would be seen in benign abnormalities, such as benign prostatic hyperplasia (BPH) and prostatitis [26]. Therefore, DCE-MRI should not be used alone but rather in conjunction with T2WI and DWI to further confirm the presence of a suspicious lesion. Limitations of DCE-MRI revolve around the differentiation of signals between PCa and prostatitis or highly vascularized BPH in the PZ and TZ, respectively, as well as lesion resolution issues due to rapid imaging [26]. 

As stated above, image optimization is crucial to providing a clinician with the best possible snapshot of a patient’s prostate lesions. The modalities described previously offer new and advanced methods for imaging the prostate. However, mpMRI had a difficult road towards full adaptation because there had never been a standardized image reporting system that defined specific guidelines for image creation and data reporting. The American college of Radiology and the European Society for Urogenital Urology convened together and made high-quality mpMRI recommendations that led to improved detection and diagnosis of clinically significant prostate cancer. This guideline is now known as PI-RADS v2 or Prostate Imaging Reporting and Data System version 2. It was modeled after the Breast Imaging Reporting and Data System (BI-RADS) for breast cancer and is a Likert-like scale aimed at helping clinicians characterize prostate lesions found on mpMRI [27].

MpMRI imaging combined with the PI-RADS v2 scoring system provides prostate imaging that increases the detection and localization of clinically significant prostate lesions (intermediate/high risk PCa) while at the same time lowering the likelihood of overdetection of clinically insignificant lesions (low risk PCa) that would otherwise lead to overtreatment and the potential for increases in unnecessary risks for patients. MpMRI was found in one study to aid in risk stratification of PCa based on lesion size, extent, and ADC value due to its superior diagnostic capabilities [28]. Another study indicated that mpMRI sensitivity exceeded 80% for detecting 0.2 cm3 of Gleason 4 + 3 or greater and 0.5 cm3 of Gleason 3 + 4 or greater disease [29]. Yet another study by Yoshizako, et al. demonstrated that the combined use of DW, DCE, and T2-weighted MRI increased accuracy in the detection of transition zone cancer compared to T2WI alone from 64% to 79% [30]. Although the optimal diagnostic results come from the combination of DW, DCE, and T2W imaging within mpMRI, the imaging modality still requires a prostate biopsy to confirm the presence of PCa and to directly access the malignancy/Gleason score of the lesion. mpMRI targeted biopsies are an area of PCa diagnostics where mpMRI has great potential to assist.

Traditionally, prostate biopsies were performed using a transrectal ultrasound-guided biopsy (TRUS-GB). Since the introduction of mpMRI in the detection and stratification of PCa, much research has been performed to elucidate the benefit of using mpMRI for targeted biopsy of suspicious prostate lesions. The main MRI-guided techniques include visual estimation, software co-registered, and in-bore MRI-guided biopsy. Visual estimation relies heavily on user comfort and the ability to use static images, thereby foregoing real-time feedback, in order to guide a prostate biopsy. Visual estimation has the advantage of lower costs and can, therefore, be a cheaper alternative for clinicians and patients. The effectiveness and accuracy of visual estimation over regular systematic TRUS varies amongst studies with the detection rate being tied heavily to user experience with the technique [31]. Other techniques, such as software co-registered biopsy and in-bore MRI biopsy, have the advantage of allowing the user to have reproducible and real-time imaging of the lesion during the biopsy process. Although more expensive and requiring great upfront investment in equipment, these methods have shown statistically significant advantages over systematic TRUS in multiple studies. These biopsy methods also have the advantage of detecting cancer in patients with previously normal biopsy results [32]. For example, in a study involving 265 patients with previously normal biopsies, there was a cancer detection rate (CDR) of 41% with 87% of those lesions being clinically significant [33].

All of these methods have shown the potential to overcome the limitations of traditional biopsy methods; namely, false-negative biopsies (undersampling) where the normal 12 biopsy cores miss the cancer completely, false risk stratification (undersampling) where clinically significant lesions are classified as low-grade due to sampling a small portion of the lesion, and identifying clinically insignificant tumors incidentally (oversampling), which leads to overtreatment [26]. In addition to MRI-targeted biopsy being useful in detecting cancers in patients with previously negative biopsies, MRI-targeted biopsies also aided in a better cancer detection rate than traditional systematic biopsy in men with no previous biopsy and aided improved risk stratification/cancer grading in patients with previously diagnosed low-grade lesions. One study showed a CDR of 70.1% in a group of 890 patients with MRI-detected lesions who underwent MRI-targeted and systematic biopsy compared to only 13.1% of 558 patients who did not have MRI-detected lesions of targeted biopsy and only underwent systematic biopsy [26]. Additionally, in a group of 141 patients, the MRI-targeted biopsy had a CDR of 90.1% in the anterior portion of the prostate, which is an area mostly missed by systematic biopsies [34]. In terms of upgrading previously low-risk lesions, a study found that 20% of a group of 388 patients had their cancers upgraded through the use of mpMRI and confirmatory visual estimation co-registration biopsy [35]. Despite the success of MRI-targeted biopsy, further studies need to be conducted to justify the cost of these methods as well to develop a standardized protocol for targeting methods.

Patients with low-risk prostate lesions can also become candidates for active surveillance, formerly known as "watchful waiting". Active surveillance is the practice of closely watching a suspicious cancer lesion and monitoring any changes/progression over time. Active surveillance aims to provide the patient with care and close monitoring while reducing the probability of overtreatment. For example, a patient’s active surveillance could consist of a doctor’s visit every six months where prostate specific antigen (PSA) levels are obtained and a digital rectal exam (DRE) is performed with the option of obtaining a biopsy every year. A physician can then make a note of any changes in the lesion and discuss treatment options with the patient. MpMRI imaging of the prostate has an important and still yet to be a fully defined role in helping to determine a patient’s eligibility for active surveillance. The risks of putting a patient on active surveillance stem from underestimating the severity of the PCa, leading to possible delays in potentially life-saving treatments. Therefore, the question was asked whether mpMRI could help properly risk stratify and monitor suspicious lesions well enough to determine which patients can enter/remain in active surveillance and which patients need to have their PCa upgraded and receive treatment. The goal is also to reduce the need for repeat biopsies, which come with their own inherent risks and limitations, as previously discussed. The Vargas study looked at 388 patients who had clinically low-risk PCa and compared the results of the endorectal MRI that was performed before the confirmatory biopsy. The study aimed to elucidate any benefit from the imaging on risk stratification between clinically significant and clinically insignificant PCa. As previously stated, the study showed an upgrade in 79 patients’ Gleason scores to clinically significant lesions. Moreover, the study showed a high negative predictive value (0.96-1.00) and specificity (0.95-1.00) for upgrading on MRIs indicating low risk lesions when confirmatory biopsy was performed, as well as high sensitivity (0.87-0.98) for upgrading when the MRI indicated that a high-risk lesion was present and then confirmed on biopsy [35]. Another study stated that “A positive MRI makes the identification of clinically significant disease at repeat biopsy more likely, especially when biopsies are targeted to suspicious MRI lesions” [36]. Additional and more robust prospective studies need to be conducted to fully determine the role of MRI in active surveillance, but the future of minimally invasive PCa monitoring and diagnosis is promising.

Multiparametric MRI is developing an integral role in the diagnosis and surveillance of PCa whether it be through lesion detection or by assisting in targeted biopsies. Further studies will confirm the ability of mpMRI to decrease false negatives/undersampling as well as limit oversampling and treatment. Confirmatory results will provide patients and physicians with alternative and less invasive methods for confronting PCa.

### MRI-TRUS

MRI-TRUS fusion techniques include (1) in-bore MR-guided biopsy (2) cognitive registration, and (3) software registration-based fusion. In-bore MR-guided biopsy uses pre-biopsy MRI images to identify lesions of interest. Subsequently, the patient will undergo biopsy within the bore of a magnet. Using MR fluoroscopy, successive images are taken to ensure that the needle is placed in the desired location and a biopsy is taken and registered. Cognitive registration entails the usage of a pre-biopsy MRI to create a map of the prostate. The physician will then incorporate these imaging findings while performing the biopsy. Although the physician is relying only on the ultrasound for real-time imaging at the time of the biopsy, the physician has a cognitive awareness of the general locations of the high-risk lesions. Software-based platforms combine a pre-biopsy MRI image with prostate contouring. At the time of biopsy, a typical 2D TRUS is obtained and sent to the fusion platform where the platform will create a 3D model using the previous MRI image and ultrasound. A live guidance of the biopsy, known as “tracking”, is thus created [37-38].

Within software-based platforms lies three major technologies: Mechanical position encoders, such as the Artemis (Eigen, Grass Valley, California, USA), the Urostation device (Koelis, LaTronche, France), and electromagnetic tracking, such as the UroNav (Philips Electronics, Amsterdam, the Netherlands). The Artemis tracks and records biopsy sites live and allows the operator to return to a positive core biopsy site (within 1.2 - 3 mm accuracy) [39]. The return to a prior site may allow for real-time pathology results and resampling. The Artemis was also three times more likely to detect cancer in patients with active surveillance or prior negative TRUS biopsy than a standard biopsy (21 vs 7 %) [40]. The Urostation device has the ability to track each core biopsy in real time. As the physician takes core biopsies, the model of the prostate is updated to reflect the biopsied regions; this allows for retrospective targeting and real-time elastic registration. The UroNav has also performed very well and has been extensively studied since its FDA approval in 2006. A landmark trial by Siddiqui, et al. showed that UroNav detected 30% more high-risk cancers (Gleason score ≥ 4 + 3) than standard TRUS biopsy [41]. 

The magnetic resonance imaging with transrectal ultrasound (MRI-TRUS) technique is the newest of common techniques to detect prostate cancer (PCa). It holds value for patients that have newly detected elevated levels of prostate-specific antigen (PSA). MRI can be used now to assess risk, increase accuracy in evaluation of tumor margins, and also guide the biopsy portion of PCa detection. The imaging used typically involves T2-weighted imaging with at least two other nuclear modalities (such as diffusion-weighted or perfusion imaging), depending on the clinical situation [26].

The MRI-TRUS technique was shown to reduce the number of biopsies done on patients that have low-grade PCa, reducing the overall diagnoses of low-grade prostate cancers, and increasing the detection of the intermediate and high-risk subgroups of patients compared to traditional modalities [26, 42]. Specifically, with intraprostatic lesions, the MRI-TRUS has also been found to be more accurate in biopsy targeting [38].

Past studies compiled and analyzed by Bjurlin, et al. showed consistently that while more cases are detected through the systematic approach, a significant portion of them was either too small or was too low-grade to be clinically significant. When MRI modalities were added, it decreased the number of unnecessary biopsies performed.

Pokorny, et al. found in a prospective study that when multiphasic magnetic resonance imaging and magnetic resonance-guided biopsy was added for PCa detection in men with elevated PSA, the need for biopsy was decreased by 51%, the diagnosis of low-risk PCa decreased by 89.4%, and the detection of intermediate and high-risk PCa increased by 17.7%, all when compared to transrectal ultrasound-guided biopsy. They found that the limitation of this study was the lack of long-term follow-up [42].

In a prospective study done by Okoro, et al. in 2015, 50 patients underwent both TRUS (systematic 12-core approach) and MRI-TRUS (targeting approach), intraprostatic lesions were better targeted by the latter during biopsy [38]. The highest percentage core involvement in the systematic approach and targeted biopsy was 22.5 ± 22 and 34.6 ± 27.4, respectively, while also finding a larger PCa tumor length, 0.33 ± 0.32 for systematic 12-core approach and 0.49 ± 0.43 for targeted MRI-TRUS targeting.

PI-RADS v2 guidelines are in place for the use of MpMRI; however, there are no current guidelines for the fusion of these modalities. Trials are underway to evaluate the accuracy of the MRI-transrectal ultrasound fusion and to reveal sources of error. A study by Wegelin, et al. used three prostate models to perform 27 biopsies using the MRI-TRUS fusion technique [43]. They found that a lesion with a 5 mm diameter in the transversal plane could be accurately sampled in 96% of cases using a single core biopsy and lesions > 3 mm can be accurately targeted. Unfortunately, due to the third dimension (3D) of depth insertion in the 3D plane, there is a remarkably larger overall error in a 3D plane in comparison to a transversal plane with errors of 2.33 mm in a transversal plane and 5.42 mm in a 3D plane. Studies such as these will ensure improvement in mapping techniques and provide a standardization in which multiple platforms may be tested and improved because they will be able to hold these methods accountable for increased overall error. 

## Conclusions

The TRUS biopsy procedure is highly user-dependent and subject to sampling errors in comparison to mpMRI or fusion modalities. These biopsies typically lead to undersampling of hard-to-reach regions, such as the central or anterior zone. Even with augmented numbers of biopsies, there is very little evidence for an increase in patient benefit and the sensitivity of detection of cancer still remains low. MpMRI, on the other hand, is able to differentiate between the different anatomical zones of the prostate and has a good visualization of the anterior zone where some cancers may arise and are not easily felt on DRE or accessed with TRUS.

MpMRI has better tissue resolution and is able to compare relative signal intensity within the prostate, allowing for regions of homogeneously low intensity (associated with higher Gleason scores) to be targeted. MpMRI can also identify lesions with poorly defined or irregular borders, which may carry a higher suspicion for cancer. With the added benefits of imaging clarity in MpMRI and standardization with the PI-RADS v2 guidelines, urologists may be more likely to find foci of high-grade tumors and less likely to miss aggressive tumors. All of these methods have shown the potential to overcome the limitations of traditional biopsy methods, namely, false-negative biopsies, false risk stratification, and identifying clinically insignificant tumors incidentally.

Multiparametric MRI is developing an integral role in the diagnosis and surveillance of PCa, whether it be through lesion detection or by assisting in targeted biopsies. The accuracy of mpMRI to correctly identify high-risk cancer and not miss significant lesions may even suggest a place for mpMRI as a surgical planning tool to determine the surgical plan or approach.

As evidenced in the literature thus far, the MRI-TRUS technique is shown to reduce the number of biopsies performed on patients that have low-grade PCa, reducing the overall diagnoses of low-grade prostate cancers and increasing the detection of the intermediate and high-risk subgroups of patients compared to traditional modalities. Fusion modalities allow the user to incorporate real-time ultrasound findings with MRI integration. It has been proven by study after study to improve the detection of clinically significant PCa. In light of these studies, we believe the less invasive MpMRI is an accurate and reliable way to diagnose and monitor prostate lesions. 
